# Importance of Genetic Polymorphisms in *MT1* and *MT2* Genes in Metals Homeostasis and Their Relationship with the Risk of Acute Pancreatitis Occurrence in Smokers—Preliminary Findings

**DOI:** 10.3390/ijms22115725

**Published:** 2021-05-27

**Authors:** Milena Ściskalska, Monika Ołdakowska, Halina Milnerowicz

**Affiliations:** Department of Biomedical and Environmental Analyses, Faculty of Pharmacy, Wroclaw Medical University, 50-556 Wroclaw, Poland; monika.oldakowska@umed.wroc.pl (M.O.); halina.milnerowicz@umed.wroc.pl (H.M.)

**Keywords:** cadmium, copper, zinc, metallothionein, single-nucleotide polymorphism, rs11640851, rs964372, rs10636

## Abstract

This study was aimed at evaluating the changes in metallothionein (MT) concentration in the blood of patients with acute pancreatitis (AP) and healthy subjects, taking into account the extracellular (plasma) and intracellular (erythrocyte lysate) compartments. The impact of single-nucleotide polymorphisms (SNPs) in the *MT1A* (rs11640851), *MT1B* (rs964372) and *MT2A* (rs10636) genes on MT concentration and their association with the concentration of metals (Cu, Zn, Cd) and ceruloplasmin as Cu-related proteins were analyzed. The concentration of a high-sensitivity C-reactive protein (hs-CRP) and IL-6 as markers of inflammation, and malonyldialdehyde (MDA), superoxide dismutase (SODs) activity and the value of total antioxidant capacity (TAC) as parameters describing the pro/antioxidative balance were also assessed. In the AP patient groups, an increased MT concentration in erythrocyte lysate compared to healthy subjects was shown, especially in individuals with the GG genotype for rs964372 in the *MT1B* gene. A Zn concentration was especially decreased in the blood of smoking AP patients with the AA genotype for SNP rs11640851 in the *MT1A* gene and the GC genotype for SNP rs10636 in MT2A, compared to non-smokers with AP, which was accompanied by an increase in the value of the Cu/Zn ratio. The exposure to tobacco smoke xenobiotics increased the risk of AP occurrence in subjects with the CC genotype for SNP rs11640851 in the *MT1A* gene by more than fourfold. The investigated polymorphisms, rs11640851 in the *MT1A* gene, rs964372 in the *MT1B* gene and rs10636 in the *MT2A* gene, seem to be an important factor in maintaining homeostasis in an organism under oxidative stress conditions.

## 1. Introduction

The availability of essentials metals, such as Zn and Cu, and their homeostasis are extremely important to the maintenance of proper functioning in the pancreas [1]. Zn is involved in a multitude of catalytic, structural and signaling processes within the pancreas [1,2]. The pancreas, as an organ with a low antioxidant potential, is particularly sensitive to the change in availability of essentials metals (Cu, Zn) and the excess of free radicals [3,4]. Decreased Zn concentration, accompanied by increased Cu concentration with prooxidative potential, is associated with numerous diseases, including pancreatitis, cancer and metabolic diseases [1,2].

Cellular homeostasis of Zn and Cu is tightly regulated by metallothioneins (MTs), which can control the influx, efflux and redistribution of these metals [2]. Metallothioneins (MTs) are low-molecular-weight (0.5–15 kDa) proteins characterized by high cysteine content (up to 30% of the amino acid residues) and are highly adept at binding metal ions and scavenging free radicals [5,6]. The metal-binding domain of MTs consists of 20 cysteine residues juxtaposed with Lys and Arg, arranged in 2 thiol-rich sites called the α- and β-domains [7]. Up to 18 metals are able to associate with MT, but Zn^2+^ is only able to be displaced by Cu^+^, Cd^2+^, Pb^2+^, Ag^+^, Hg^2+^ and Bi^2+^ [8]. The C-terminal part (α-domain) is capable of binding 4 divalent metal ions (especially Zn^2+^ or toxic Cd^2+^) through 11 cysteine residues. However, the N-terminal part, marked as the β-domain, is able to coordinate up to three divalent ions (Zn^2+^ or Cd^2+^) through nine cysteine residues [7]. The α-domain has a higher affinity for Zn^2+^ than the β-domain [9]. The β-domain seems to be a domain preference for Cu^+^ ions binding, while the α-domain has been associated with a preference for Zn^2+^ or Cd^2+^ coordination [10,11]. In the case of exposure to toxic metals such as Cd^2+^, MT could coordinate both the sequestration of the toxic metal and the release of a Zn^2+^ ion [6]. The release of Zn^2+^ ions from the MT molecule begins from a more labile β-domain, and this process limits the availability of MT to perform its function as a donor of Zn^2+^ ions for signaling processes [9].

MTs genes, located in chromosome 16 (16q12-22), are tightly linked, and they consist of eleven MT1 genes at a minimum (MT-1A, -B, -E, -F, -G, -H, -I, -J, -K, -L, and -X), and one gene for the *MT2A* gene [7]. MT-1 and MT-2 are the most widely distributed MT isoforms. MT-1 and MT-2 are expressed in many tissues, and at a particularly high level in the liver and kidney [12]. Our earlier study showed a moderate immunohistochemical reaction in pancreatic islets and a strong, focal MT expression in acinar cells in the patient with chronic pancreatitis [13]. It is known that exposure to metals (Zn, Cu, Cd, Hg,) can influence an increase in MT gene transcription through metal response, element-binding transcription factor-1 (MTF-1) or cis-acting DNA elements [7]. Additionally, Cd, a major toxic metal included in tobacco smoke, can impair Zn and Cu homeostasis and substitute Zn or Cu in the MT molecule, which can limit the bioavailability of these metals to metal-binding proteins, such as ceruloplasmin (Cp) or metal-related enzymes, e.g., Cu/Zn superoxide dismutase (Cu/Zn SOD) (14). Besides metal exposure, there are a range of the factors, such as oxidative stress, a diet rich in Zn, proinflammatory cytokines and glucocorticoids, that interact directly or indirectly with regulatory elements of the MT gene and increase gene transcription [14,15]. Differences in the gene expression of MT-1 and MT-2 isoforms in the human body were noticed. The difference between MT-1 and MT-2 is also associated with their half-lives, which can influence the rate of MT degradation [16]. It is known that the MT-2 level in the liver is three times higher than MT-1 [17]. This means that MT-1 can be more susceptible than MT-2 to intracellular degradation, depending on environmental factors [18].

It was found that the expression of MT genes and the functioning of MT isoforms may also be influenced by internal factors, such as single-nucleotide polymorphisms (SNPs) [5]. The association of SNPs in MT genes with various pathological conditions in the pancreas, such as carcinogenesis and inflammatory diseases, was described [19,20,21]. It was shown that SNP rs11640851 in the *MT1A* gene is related to the development of type 2 diabetes [20,22]. According to the NCBI database, this SNP is associated with the change from cytosine (C) to adenine (A), resulting in the substitution of threonine (ACC) to asparagine (AAC) in the polypeptide chain of MT1A. It was noted that the CC genotype was associated with higher zinc release by MT, reduced MT levels and low IL-6 plasma concentrations [5,22]. However, SNP rs964372 in the *MT1B* gene was associated with the decreased utilization of fatty acids in type 2 diabetes patients [20]. Furthermore, Zavras et al. showed that genotype CC for rs9643722 in the *MT1B* gene could contribute to protection against oral squamous cell carcinoma [23]. In the human body, the *MT2A* gene shows the strongest expression among MT isoforms (about 50% of total MT expression) [24]. However, SNP rs10636, located in the *MT2A* gene, was related to blood Cu, Zn and Cd concentration and inflammatory cytokines levels [5].

Though the association of the above-mentioned SNPs in MT genes with metal concentrations and inflammatory diseases was shown, no studies have addressed the role of these SNPs in the course of acute pancreatitis (AP). Therefore, the aim of this study was to investigate the SNPs that may contribute to the exacerbation of inflammation, rs11640851 in the *MT1A* gene, rs964372 in the *MT1B* gene and rs10636 in the *MT2A* gene, in patients with acute pancreatitis (AP) and healthy subjects. The study aimed to assess the influence of the above-mentioned SNPs on the concentration of MT in the extracellular (plasma) and intracellular (erythrocyte lysate) compartments. In this study, the concentration of MT-related metals (Cu, Zn) Cp and the total activity of superoxide dismutase (SODs) as metal-dependent proteins in regard to the genotype for rs11640851 in the *MT1A* gene, rs964372 in the *MT1B* gene and rs10636 in the *MT2A* gene were measured. The above-mentioned parameters were analyzed in terms of tobacco smoke exposure, the intensity of which was assessed by the Cd and cotinine concentrations in the blood. The concentration of a high-sensitivity C-reactive protein (hs-CRP) and IL-6 as markers of inflammation, and malonyldialdehyde (MDA) and the value of total antioxidant capacity (TAC) as parameters describing the pro/antioxidative balance were also assessed.

## 2. Results

### 2.1. The Concentration of Metals (Cu, Zn, Cd), Metallothionein, Selected Parameters of Pro/Antioxidative Balance and Inflammatory Markers in the Groups of AP Patients and Healthy Subjects

In the examined groups, a significant increase in concentrations of inflammatory markers, hs-CRP and IL-6 in the blood of AP patients compared to healthy subjects was observed (*p* < 0.0001 for both parameters). A 3-fold increase in the MDA concentration and about a 30-fold increase in the value of TAC in the blood of AP patients compared to healthy subjects were noted (MDA: *p* < 0.0001 for smokers and non-smokers, TAC: *p* < 0.0001 for non-smokers and *p* = 0.0002 for smokers) (Table 1).

Though there were no differences in Cu concentration between the group of AP patients and healthy subjects, an elevated value in the Cu/Zn ratio in the group of AP patients compared to healthy subjects was shown (*p* < 0.0001 for smokers and non-smokers). An almost threefold increase in MT concentration in the erythrocytes of AP patients compared to healthy subjects was observed (*p* < 0.0001 in both examined groups, smokers and non-smokers). This alteration was not noted in plasma MT concentration. In the group of AP patients, more than a twofold increase in the SODs activity in erythrocytes compared to healthy subjects was found (*p* < 0.0001 in both examined groups, smokers and non-smokers). It was shown a decreased Cp concentration in the group of AP patients, compared to healthy subjects (*p* < 0.0001 in both examined groups).

An increased Cd concentration in the smoker groups of both healthy subjects and AP patients, compared to non-smokers, was observed. No differences in the concentration of Cu and Zn were shown between smoking and non-smoking healthy subjects. However, in the group of smoking AP patients, a decreased Zn concentration compared to non-smokers in this group was noted (Table 1).

It was analyzed the concentrations/activities of above mentioned parameters in the blood of AP patients with regard to etiology of disease, but no differences were shown (Supplementary Table S1). 

Intersexual variability in the concentration of metals, MT and the above-mentioned parameters of pro/antioxidative balance were analyzed. A decreased Zn concentration and an increase in the value of the Cu/Zn ratio in the group of healthy subjects were observed. No differences in the above-mentioned parameters between women and men were observed in the group of AP patients (Supplementary Table S2).

### 2.2. Results of Genotyping

The MT concentration and the parameters related to metal concentrations (Cu, Zn, Cd) examined in this study were analyzed for the single-nucleotide polymorphism (SNP) rs11640851 in the *MT1A* gene, rs964372 in the *MT1B* gene and rs10636 in the *MT2A* gene. The results of PCR products after amplification for SNPs were seen in all samples. After the restriction enzyme reaction, the target nucleotide sequences were seen for all genotypes for studied SNPs. The identified genotypes were labeled according to the presence or absence of the enzyme restriction site for the examined SNPs. The frequency of genotype occurrence for the above-mentioned SNPs in the study population is presented in Table 2.

Heterozygous CA was the most common genotype for SNP rs11640851 in the *MT1A* gene in both AP patients and healthy subjects. It was noted that the frequencies of the occurrence of AA and CC genotypes were similar in the groups of healthy subjects. However, it was found that the CC genotype appeared the least often among the AP patients. It was noted that heterozygotes were the most common genotypes for SNP rs964372 in the *MT1B* gene and rs10636 in the *MT2A* gene. The CC genotype for rs964372 in the *MT1B* gene and the GG genotype for rs10636 in the *MT2A* gene appeared the least often among the study population (Table 2).

### 2.3. The Concentration of Metals (Cu, Zn, Cd), Metallothionein, Selected Parameters of Pro/Antioxidative Balance and Inflammatory Markers in Context of Genotypic Variability of Single-Nucleotide Polymorphisms rs11640851 in the MT1A Gene

In the group of non-smoking healthy subjects with the CA genotype, it was shown that the value of the Cu/Zn ratio was significantly higher compared to individuals with the CC genotype (*p* = 0.0404) (Table 3). In the group of non-smoking AP patients with the CA genotype, the highest value of the Cu/Zn ratio was shown. It was accompanied by the highest Cp concentration, which was statistically significant in individuals with the AA genotype (*p* = 0.0115) (Table 3).

An increase in Cu concentration in the group of smoking AP patients with the CA genotype compared to non-smokers was also found (*p* = 0.0376). In the blood of smoking AP patients with the AA genotype, a decreased Zn concentration compared to the non-smoking individuals with this genotype was shown (*p* = 0.0338). Moreover, in the AP patient group with the AA genotype, the Cp concentration was significantly higher in smokers compared to non-smokers (*p* = 0.0092). The TAC value was lower in smoking AP patients with CA (*p* = 0.0376), AA and CC (not statistically different) genotypes, compared to non-smokers with AP (Table 4).

In both groups, healthy subjects and AP patients, an increased Cd concentration in the blood of smokers compared to non-smokers was shown. This difference was noted in the individuals with each of the genotypes: CA, AA and CC (*p* = 0.0048 *p* = 0.0404 and *p* = 0.0367 in the group of healthy subjects, respectively, and *p* = 0.0339, *p* = 0.0062 and *p* = 0.0489 in the group of AP patients, respectively) (Table 3 and Table 4).

### 2.4. The Concentration of Metals (Cu, Zn, Cd), Metallothionein, Selected Parameters of Pro/Antioxidative Balance and Inflammatory Markers in Context of Genotypic Variability of the Single-Nucleotide Polymorphism rs964372 in the MT1B Gene

In the group of healthy subjects, a significantly higher Cd concentration in smokers with CG, GG and CC genotypes compared to non-smokers was shown (*p* = 0.0027, *p* = 0.0074, *p* = 0.0143, respectively). We also noticed an increased Zn concentration in healthy smokers with the CC genotype compared to non-smokers (*p* = 0.0455) (Table 5).

In the AP patient group with CG and GG genotypes, increased Cd concentrations in erythrocyte lysate in smokers compared to non-smokers (*p* = 0.0143, *p* = 0.0330 respectively) were shown. Moreover, in the AP patient group with the GG genotype, the MT concentration in erythrocyte lysate was significantly higher in smokers compared to non-smokers (*p* = 0.0281). In the erythrocyte lysate of smoking AP patients with the GG genotype, an increased MT concentration compared to the smoking AP patients with the CC genotype was shown (*p* = 0.0217) (Table 6).

In the group of non-smoking AP patients with the GG genotype, the value of the Cu/Zn ratio was significantly higher in comparison with the individuals with the CG genotype (*p* = 0.0477). In the group of smoking AP patients, the difference in the value of the Cu/Zn ratio was not shown, but a decrease in Zn concentration (*p* = 0.0275) and an increase in Cp concentration (*p* = 0.372) in the blood of smoking AP patients with the CG genotype compared to non-smokers with this genotype were noted (Table 6).

### 2.5. The Concentration of Metals (Cu, Zn, Cd), Metallothionein, Selected Parameters of Pro/Antioxidative Balance and Inflammatory Markers in Context of Genotypic Variability of Single-Nucleotide Polymorphisms rs10636 in the MT2A Gene

In the group of healthy subjects with the GC genotype, the concentrations of MT and Cd in erythrocyte lysate were significantly higher in smokers compared to non-smokers (*p* = 0.0219 and *p* < 0.0001, respectively). In the blood of non-smoking healthy subjects with the CC genotype, a threefold increase in MDA concentration compared to individuals with the GC genotype was shown (*p* = 0.0100). A similar change in MDA levels was noticed in the blood of non-smoking healthy subjects with the GG genotype compared to the GC genotype, but this difference was not statistically significant (Table 7).

In the smoking AP patients with the GG genotype, a significantly higher concentration of MT and SODs activity in erythrocyte lysate compared to individuals from this group with the GC genotype was shown (*p* = 0.0275, *p* = 0.0331, respectively). In the blood of smoking AP patients with the GC genotype, an increase in Cd concentration compared to non-smokers with this genotype was found (*p* = 0.0019) (Table 8).

An increase in Cu concentration (*p* = 0.0015), a decrease in Zn concentration (*p* = 0.0017) and an increase in the value of the Cu/Zn ratio (*p* = 0.0253) in the group of smoking AP patients with the GC genotype compared to non-smokers with this genotype were shown. Moreover, a decrease in Zn concentration in the blood of smoking AP patients with the GC genotype in comparison to individuals with CC genotype was observed (*p* = 0.0388) (Table 8).

### 2.6. Results for the Odds Ratio Analysis and the Correlation Coefficients

In this study, we examined the association between AP occurrence and exposure to tobacco smoke in subjects with the genotypes for SNPs rs11640851, rs964372 and rs10636 in the *MT1A, MT1B* and *MT2A* genes. In the group of smokers with the CC genotype for SNP rs11640851 in the *MT1A* gene, the risk of AP recurrence was elevated by more than four times (OR = 4.5000, *p* = 0.0354). In the case of CA and AA genotypes, this association was not statistically significant (OR = 2.0979, *p* = 0.1676 and OR = 0.4934, *p* = 0.2838, respectively).

The association between AP occurrence and smoking in the subjects with CC, GC and GG genotypes for SNP rs964372 in the *MT1B* gene was not observed (OR = 1.7143, *p* = 0.4702; OR = 1.6765, *p* = 0.3579; OR = 1.6410, *p* = 0.4062, respectively). Similarly, the association between AP occurrence and smoking in the subjects with GG, GC and CC genotypes for SNP rs10636 in the *MT2A* gene was not found (OR = 0.9959, *p* = 0.9998; OR = 1.4999, *p* = 0.3176; OR = 2.5000, *p* = 0.3019, respectively).

Statistically significant correlations in terms of genotype for SNPs, rs11640851 in the *MT1A* gene, rs964372 in the *MT1B* gene and rs10636 in the *MT2A* gene, were noticed, as presented in Supplementary Tables S3–S5.

## 3. Discussion

Impaired zinc and copper homeostasis and an increase in the value of the Cu/Zn ratio significantly contribute to the formation of free radicals, which are considered to be one of the factors involved in the pathogenesis of acute pancreatitis [14,25]. A pro/antioxidant imbalance leads to disorders of cell signaling pathways [26,27], which manifests in an increase in proinflammatory cytokine expression [28]. MT plays an important role in the maintenance of Cu and Zn homeostasis [29]. Numerous studies confirm an increase in MT concentration as a response to inflammation [30,31,32]. There are numerous reports on the relationship between the polymorphism of genes encoding MT and the concentration of metals in the body [29,33,34,35,36]. To date, the above relationships have not been studied in the group of patients with acute pancreatitis.

Our previous study showed a significant decrease in Zn concentration in the group of patients with pancreatic diseases compared to healthy subjects [14], which was also confirmed in this study. The Zn deficiency observed in the blood of patients with pancreatitis can be associated with the low supply of this metal, especially in people aged ≥60, and lifestyle (smoking, alcohol abuse) [37]. In this study, a decrease in Zn concentration accompanied by an increase in the value of the Cu/Zn ratio in AP patients could contribute to the intensification of oxidative stress, which can be confirmed by elevated MDA concentration as a byproduct of lipid peroxidation [25,38].

An increase in MT concentration in erythrocyte lysate in both non-smoking and smoking groups of AP patients compared to healthy subjects observed in our study may be the result of intensive oxidative stress accompanying the inflammatory process. Inflammation contributes to the induction of metallothionein synthesis, which is highly adept at scavenging free radicals [39,40]. Additionally, our results showed increased total activity of SODs in erythrocytes of AP patients compared to healthy subjects, which could be related to increased MT concentration. MT, as a donor of Zn2+ ions, can take part in antioxidative defense and can regulate the activity of superoxide dismutase, especially its SOD1 and SOD3 isoenzymes [41]. The activity of Cu/Zn SOD strictly depends on metals such as Zn and Cu, where MT participates in its metabolism [14]. Therefore, the increased MT concentration in erythrocytes indicates an important antioxidative role of this protein in the course of acute pancreatitis. This is confirmed by our previous studies, in which an increased expression of MT in pancreatic tissues in the course of chronic pancreatitis was shown. This suggests that this protein plays an essential role in maintaining the homeostasis of the pancreas and may be a specific marker of pancreatitis [13].

Aside from acute pancreatitis, exposure to tobacco smoke xenobiotics is an additional factor in disturbing Zn and Cu homeostasis and the major factor in increasing the risk of the development of this disease. As a result of tobacco smoking, the organism accumulates Cd in erythrocytes, which have pro-oxidative and proinflammatory effects [26]. This study showed a fivefold increase in Cd concentration in erythrocyte lysate of smokers compared to non-smokers in the AP patient group, which was accompanied by an increase in MT concentration in erythrocyte lysate. Tobacco smoking, and an associated elevation in Cd concentration, can induce MT synthesis as a protein taking part in the detoxification of this metal [42].

There are reports showing the influence of SNPs in the gene encoding of MT on its concentration in blood and a change in the functionality of this protein. SNPs in MT genes may be a factor for determining differences in individual sensitivity to the effects of free radicals and heavy metals. The occurrence of polymorphisms may be related to the intensification or the appearance of pathophysiological processes in the body, such as cancers or inflammation, but there are no studies dealing with acute pancreatitis.

### 3.1. The Influence of SNP rs11640851 in the MT1A Gene on the Concentration of Metallothioneins, Metals and Selected Parameters of Pro/Antioxidative Balance

While this study did not show an association of MT concentration with the genotype for rs11640851, changes in metal concentrations in the blood were noted. It was observed that the **AA genotype** for SNP rs11640851 in the *MT1A* gene can elevate the susceptibility of healthy people to the toxic effects of tobacco smoke xenobiotics, which reflects the highest concentration of Cd in the blood of people in this group. Similar changes were observed in the blood of smoking patients with CA and AA genotypes. In addition, a decrease in Zn concentration in the group of smoking AP patients with the AA genotype compared to non-smokers was observed. These results may indicate that people with the AA genotype are most sensitive to the harmful effects of tobacco smoke xenobiotics.

Although SNP rs11640851 in the *MT1A* gene contributes to changes in Cd concentration in the group of smoking AP patients with CA and AA genotypes, MT concentration and total SOD activity in erythrocyte lysate and plasma remained unchanged. On this basis, it can be assumed that the occurrence of CA and AA genotypes for this SNP does not affect MT molecule synthesis but may contribute to changes in MT function, which affects Zn homeostasis, especially in the group of smoking AP patients with the AA genotype. In the present study, it was noted that smoking AP patients with the AA genotype may be more susceptible to oxidative stress, as evidenced by the highest concentration of MDA in their blood. Persistent oxidative stress may intensify inflammation in these individuals, which is confirmed by a slight increase in IL-6 concentration compared to subjects with CA and CC genotypes.

Analysis of the SNP rs11640851 in the *MT1A* gene showed the lowest concentration of Zn in the blood of patients with the **CA genotype**, in both non-smokers and smokers, compared to patients with AA and CC genotypes. Additionally, in the group of smoking AP patients with the CA genotype, the highest concentration of Cd and a significant increase in the concentration of Cu in comparison to non-smokers were found. It was also shown that an increase in the Cd concentration in the subjects with the CA genotype contributes to the disruption of Zn and Cu homeostasis, which further aggravates the decrease in Zn concentration in the blood of smoking AP patients. AP patients with the CA genotype are predisposed to the accumulation of Cd and its binding to the MT molecule, with the simultaneous release of Zn^2+^ ions from its structure. It is known that the presence of the CA genotype and the formation of a stable MT–Cd complex, by displacing Zn^2+^ from the MT–Zn complex, may limit MT’s function as a Zn donor for cellular processes such as insulin synthesis and secretion [43]. The presence of the CA genotype may contribute to the thermodynamic changes in the *MT1A* structure and the loss of its β-cluster symmetry after the binding of Cd^2+^ ions, which may be related to the weakening of the Zn^2+^ bond [6]. Therefore, it can be assumed that the MT molecule in individuals with this genotype shows an increased detoxification function while weakening its role as a zinc donor. Moreover, a decrease in TAC values in the blood of smoking AP patients with the CA genotype compared to non-smokers was noted. Low antioxidant potential in the blood of smokers may result from the consumption of antioxidants involved in the neutralization of free radicals in tobacco smoke [44].

In the examined groups of non-smokers with the **CC genotype**, of both healthy subjects and AP patients, the highest concentration of Zn was observed. The cause of the observed changes may be the influence of the CC genotype on the affinity of Zn^2+^ to the MT molecule, which may affect the modulation of Zn concentration in the blood [22]. Interestingly, in the group of smoking subjects with the CC genotype, a more than 4.5-fold increase in the risk of AP occurrence was shown. This may be related to the disorder of the molecular effect of MT as a protein taking part in distributing cellular Zn. Zinc is an intracellular mediator of apoptosis, which can interfere with the action of Ca^2+^ [45]. The excessive increase in Ca^2+^ ions in the pancreatic acinar cells can contribute to disturbances in cellular signal transduction, cell vacuolization and the activation of trypsinogen, which is a key factor in the pathogenesis of acute pancreatitis [46].

### 3.2. The Influence of SNP rs964372 in the MT1B Gene on the Concentration of Metallothioneins, Metals and Selected Parameters of Pro/Antioxidative Balance

In the group of non-smoking AP patients, it was shown that the **GG genotype** was associated with an increased value of the Cu/Zn ratio, which was statistically significant compared to AP patients with the CC genotype. In contrast, no differences in blood Zn and Cu concentrations were noted in the group of smoking AP patients with this genotype. However, it was observed that exposure to an additional factor that is an inductor of free radicals, such as smoking, causes a significant increase in MT concentration in the erythrocytes of AP patients with the GG genotype compared to those with the CC genotype. These results can confirm that exposure to tobacco smoke xenobiotics and the oxidative stress associated with it may induce MT synthesis, which is demonstrated in other studies [14]. In the group of smoking patients, the increase in MT synthesis may play a protective role against the loss of Zn from the body. Accordingly, this protein can properly fulfill its function as a Zn reservoir and donor for metalloenzymes such as SOD.

In the group of smoking AP patients with the **CG genotype**, despite a significant increase in Cd concentration, no changes in MT concentration compared to non-smoking AP patients with this genotype were observed. Moreover, in the group of smoking AP patients with the CG genotype, a significant decrease in Zn concentration compared to the group of non-smoking AP patients was shown. These results indicate that the lack of an increase in MT concentration in the course of AP and simultaneous exposure to tobacco smoke xenobiotics may result in changes in Cu and Zn homeostasis, as evidenced by a decrease in Zn concentration in subjects with the CG genotype. Additionally, a positive correlation between the value of the Cu/Zn ratio and MDA concentration in the group of AP patients with the CG genotype was demonstrated. This may confirm that the disturbance of Cu and Zn homeostasis in this group exerts a pro-oxidative effect, which manifests in damage to cell membranes and an increase in the concentration of MDA, a marker of lipid peroxidation.

### 3.3. The Influence of SNP rs10636 in the MT2A Gene on the Concentration of Metallothioneins, Metals and Selected Parameters of Pro/Antioxidative Balance

Numerous reports have confirmed the relationship between SNP rs10636 in the *MT2A* gene and the concentration of metals in the blood [5,29,47]. The studies conducted by Kayaalti et al. showed a significant decrease in the concentration of Zn in the blood of healthy people with the GG genotype for SNP rs10636 in the *MT2A* gene [36]. In this study, no differences in the concentration of Zn in the blood of healthy subjects with the GC, GG and CC genotypes were observed. However, in the group of smoking healthy subjects with the **GC genotype**, an increase in the concentration of Cd and MT in erythrocytes that was not accompanied by differences in the concentration of Cu and Zn was noted. Our study shows the relationship between the GC genotype and the disturbance of Cu homeostasis in the group of smoking AP patients, which can confirm the lowest concentration of Zn and the highest Cu and Cd level in the blood of individuals with this genotype. This may indicate a predisposition of the GC genotype to decreased Zn concentration, intensifying proinflammatory and pro-oxidative processes. The results obtained in this study confirm that Cd contained in tobacco smoke is an additional factor in exacerbating the disturbances of Cu and Zn homeostasis in the group of patients with acute pancreatitis, especially among subjects with the GC genotype.

In the group of smoking patients with AP, it was observed that the **GG genotype** was associated with the highest concentration of Zn in the blood and the lowest value of the Cu/Zn ratio compared to patients from this group with the GC and CC genotype (the difference was not statistically significant). The above results may indicate that the above-mentioned polymorphism plays an important role in the maintenance of Zn homeostasis, necessary for the proper functioning of the pancreas. In addition, in the group of smoking AP patients, it was noted that the GG genotype was associated with a significant increase in MT concentration in erythrocytes compared to non-smokers, which may contribute to faster neutralization of free radicals in the intracellular environment in AP patients with this genotype.

In summary, the course of AP is associated with the disturbance of Zn and Cu homeostasis, which results in an increased value of the Cu/Zn ratio and an intensification of the inflammatory process. The investigated polymorphisms, rs11640851 in the *MT1A* gene, rs964372 in the *MT1B* gene and rs10636 in the *MT2A* gene, seem to be an important factor in maintaining homeostasis in an organism under oxidative stress conditions. An additional factor aggravating Cu/Zn imbalance is tobacco smoke exposure, especially in individuals with the CC genotype for SNP rs11640851 in the *MT1A* gene, which is associated with a more-than-four-times higher risk of AP occurrence.

## 4. Materials and Methods

### 4.1. Subjects

**Figure 1:**
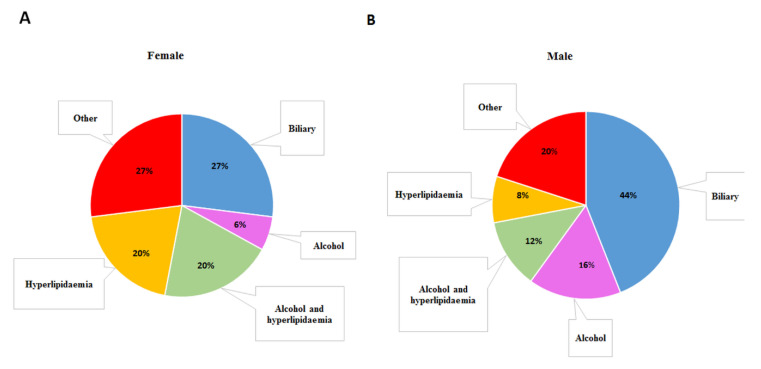
Etiology of acute pancreatitis in the group of (**A**) women and (**B**) men.

The study group included 40 patients with acute pancreatitis (17 non-smokers and 23 smokers) aged 49.4 ± 16.0 (BMI: 26.0 ± 4.4 kg/m^2^), hospitalized in the Second Department of General and Oncological Surgery, Wroclaw Medical University (Wroclaw, Poland) and 51 healthy volunteers (26 non-smokers and 25 smokers) aged 46.3 ± 8.5 (BMI: 22.9 ± 1.8 kg/m^2^), classified as the control group. All subjects provided their written informed consent for inclusion before they participated in the study. The study was conducted in accordance with the Declaration of Helsinki and approved by the Bioethics Committee of the Wrocław Medical University (No.: KB: 592/2013, KB: 529/2018 and KB: 215/2020). The patients were classified into the AP group on the basis of personal interviews, epigastric pain with touch sensitivity and medical and laboratory blood tests. Specific criteria for the inclusion of the patients in the study were described in [41]. The severity of the disease was determined according to the Atlanta classification. patients with mild AP (no organ failure, no local complications) were qualified for the study. The exclusion criteria were accompanying diseases, such as cardiovascular diseases, liver diseases, cancer, diabetes, arthritis and ongoing inflammatory states other than AP, and simultaneous treatment with more than two types of drugs, regardless of their mechanism of action. The clinical characteristics of the patients and the etiology of AP are presented in Table 9 and Figure 1, respectively. The healthy volunteers were classified into the control group on the basis of the physical examination conducted by primary care physicians, interviews, laboratory tests and the evaluation of a questionnaire. Healthy subjects with diagnosed diseases and alcohol abusers were excluded from this study.

All hospitalized patients and healthy volunteers received complete and thorough information about the study. Basic anthropometrical assessments were also performed. Personal interviews about lifestyle were carried out. The participants answered questions about their health and nutritional habits, any use of medications/dietary supplements, frequency of alcohol intake and smoking history (the number of cigarettes smoked per day, duration of smoking, smoking cessation, smoking-related disease occurrence, passive exposure to cigarette smoke). Based on elevated levels of cotinine in the serum (a nicotine metabolite) and direct personal interviews, hospitalized patients and healthy volunteers were categorized into smoking (with cotinine concentration ≥ 10 ng/mL) and non-smoking groups (cotinine concentration ≤ 10 ng/mL).

### 4.2. Materials

The material was venous blood collected in the morning after overnight fasting, up to 24 h from the onset of the first symptoms of acute pancreatitis. The blood samples of the healthy individuals were obtained from the biobank of the Polish Center for Technology Development (Wroclaw, Poland).

The biochemical analyses were performed in serum, plasma, erythrocyte lysate and DNA, isolated from whole blood, collected from patients with AP and healthy volunteers. The serum was obtained according to the standard procedure by depositing venous blood into disposable trace element-free tubes (Cat. No.: 368815, Becton Dickinson, Heidelberg, Germany) with serum clot activator, left at 25 °C to complete thrombosis and centrifuged (1.2 g/20 min). The plasma and erythrocyte lysate were obtained by collecting whole blood in tubes containing heparin (Cat. No.: 368886, Becton Dickinson, Germany). Then, the blood was centrifuged (2500 g/15 min) to separate the plasma from buffy coat and erythrocyte pellet. The samples of serum and plasma were portioned and stored in sealed tubes (Cat. No.: 0030102.002, Eppendorf, Hamburg, Germany) at −25 °C until analysis.

The erythrocyte lysate was obtained by drawing 0.5 mL of erythrocytes pellet from tubes containing heparin and washed twice in an equal volume of 0.9% NaCl. Then, the erythrocytes were lysed by the addition of 0.7 mL ice-cold, super-pure, demineralized distilled water (dilution 1:1.4). The resulting lysate was portioned and stored in sealed tubes (Cat. No.: 0030102.002, Eppendorf, Germany) at −25 °C until analysis.

The platelet–leukocyte buffy coat layer (for DNA isolation) was obtained by the centrifugation of the whole blood, deposited into tubes containing heparin (2.5 g/15 min) to separate it from plasma and erythrocyte pellet. The buffy coat was transferred to a fresh tube, resuspended and washed in PBS, then centrifuged at 16,000× *g* for 3 min to remove the PBS. The samples of the buffy coat were stored at −80 °C until analysis.

### 4.3. Methods

Cotinine concentration in serum was determined with the use of a commercial Cotinine ELISA test (Cat. No.: EIA-3242, DRG International, Springfield, NJ, USA). The test supplies qualitative screening results for cotinine in human serum at a cutoff concentration of 10 ng/mL.

Metal (Cu, Zn, Cd) concentration analysis in the blood was performed in a certified laboratory in the Atomic Absorption Spectrophotometry Laboratory at the Department and Clinic of Internal and Occupational Diseases and Hypertension in Wroclaw Medical University by atomic absorption spectrometry using a Solaar M6 spectrometer (Solaar House, Cambridge, UK). Cu and Zn concentrations in serum were determined by the flame atomic absorption spectrometry (FAAS) method in an air–acetylene flame at a wavelength of λ = 324.8 nm. The reference solutions were Single-Element Copper (Zinc) Standard 1000 g/mL certified by CPI International. Based on Cu and Zn concentration, the Cu/Zn ratio was calculated. The cadmium (Cd) concentration was measured in whole blood by graphite furnace atomic absorption spectrometry (GFAAS) in the graphite Massman cuvette, with the absorbance measurement at a wavelength of λ = 228.8 nm, with Zeeman background correction. The reference material was BCR-196 certified by the Institute for Reference Materials and Measurements.

The total concentration of MT was measured in erythrocyte lysate and plasma following the procedure developed in our laboratory, using the two-step direct ELISA method with commercial primary monoclonal antibody Clone E-9 (Cat. No: M0639, DAKO, Glostrup, Denmark) and the secondary biotinylated polyclonal Goat Anti-Mouse IgG antibody (Cat. No: E0433, DAKO, Denmark) and the standard of MT (containing isoforms MT-1 and MT-2) isolated from a human liver as described earlier [48]. The MT concentration in erythrocyte lysate was expressed as nanograms of MT per gram of hemoglobin.

The concentration of ceruloplasmin (Cp) in the blood serum was determined using the Enzyme-linked Immunosorbent Assay Kit for Ceruloplasmin (Cat. No. SEA909Hu 96. Cloud-Clone Corp., Katy, TX, USA). The concentration of plasma MDA was assayed spectrophotometrically with the use of a commercial Lipid Peroxidation (MDA) Assay Kit (Cat. No.: MAK085-1KT, Sigma-Aldrich, Steinheim, Germany). This assay is based on the reaction of MDA with thiobarbituric acid to form a colorimetric product that is adequate for the MDA concentration in the sample. The absorbance of the sample was measured at λ = 532 nm.

High-sensitivity CRP (hs-CRP) concentration in serum was assayed by the turbidimetric method with a C-reactive protein hs test (Cat. No.: 31927, Biosystems, Barcelona, Spain).

The IL-6 concentration in plasma was measured with the use of a Human IL-6 DuoSet ELISA assay kit (Cat No: DY206-05, R&D Systems, Minneapolis, MN, USA). Total SOD activity in plasma and erythrocyte lysate was measured using a commercial kit with xanthine oxidase, hypoxanthine and tetrazole salt (Cat. No.: 706002, Cayman Chemical, Ann Arbor, MI, USA). The activity of SOD in plasma was expressed as U/mL. One unit of SOD activity is defined as the amount of enzyme needed to dismutase 50% of available superoxide radicals. The activities of SOD in erythrocyte lysate were converted to grams of hemoglobin and expressed as U/g Hb.

The total antioxidant capacity (TAC) in plasma was assayed with the use of the OxiSelect™ Total Antioxidant Capacity (TAC) Assay Kit (Cat. No.: STA-360, Cell Biolabs, Inc., San Diego CA, USA). In this procedure, uric acid was applied as a reference antioxidant, and the value of TAC was demonstrated as uric acid equivalents (UAE). Moreover, the obtained TAC value was converted into copper reducing equivalents (CRE) by the following formula: TAC (µM CRE) = TAC (mM UAE) × 2189 (μM Cu^2+^)/(mM UAE), where 1 mM of UAE is equal to 2189 μM CRE.

Hemoglobin concentration in the erythrocyte lysate was measured using Drabkin’s reagent (Cat. No.: 20082, Aqua-Med, Poland).

### 4.4. Genotyping Analyses

The DNA was isolated from the platelet–leukocyte buffy coat layer using a commercial kit (Syngen Blood/ Cell DNA Mini Kit, Cat. No.: SY221012, Syngen Biotech, Wroclaw, Poland), following the manufacturer’s recommendations. DNA purity was measured using a µDrop™ Plate (Cat. No.: N12391, Thermo Fisher Scientific, Waltham, MA, USA) at λ = 260 nm. To determine the polymorphisms of MT1A (rs11640851, NC_000016.10:g.56639315C>A, NP_005937.2:p.Thr27Asn), MT1B (rs964372, NC_000016.9:g.56686030C>A, NM_005947.3:c.28+137C>A) and MT2A (rs10636, NC_000016.9:g.56643343G>C, NM_005953.5:c.*77=), the polymerase chain reaction (PCR) and restriction fragment length polymorphism analysis (PCR-RFLP) were carried out.

The primers were designed by the Primer-BLAST program (National Center for Biotechnology Information) based on gene sequences from the database GenBank (National Center for Biotechnology Information). The primers and restriction enzymes were stored at −20 °C in accordance with the manufacturer’s instructions.

The PCR reactions in the *MT1A* gene (located in exon 2 [35] in the β-domain coding region of MT molecule), *MT1B* gene (located in intron 1 [5] in the β-domain coding region of MT molecule) and *MT2A* gene (located in 3′UTR [49]) were performed with 20.0 µL as the final volume of PCR reaction mixture. It consisted of 0.6 μL of primers (working concentration: 10 pmol/μL), 2 µL extracted DNA, 12.8 µL PCR clean water and 4 µL Gold Hot-Start PCR Mix (Cat. No.: SY550231, Syngen). The initial denaturation was performed at 95 °C for 15 min and was then followed by 35 cycles within 40 s at 95 °C (denaturation), 35 s at 55–58 °C (annealing, depending on SNP; Table 10) and 45 s at 72 °C (elongation). A final elongation step was performed at 72 °C for 15 min. The PCR products were digested with restriction enzyme (Table 11) and the digested fragments were visualized in 3% agarose gel (Cat. No.: SY521011) with Green DNA Gel Stain (Cat. No.: SY521032, Syngen Biotech, Wroclaw, Poland).

### 4.5. Statistical Analysis

The data were presented as mean ± deviation and upper quartile, median and lower quartile. The normality of the variables was tested by the Shapiro–Wilk test. The differences between the examined groups were tested using the Student’s *t*-test or a nonparametric Mann–Whitney U test. Differences between the three groups were analyzed by one-way ANOVA (two-way Analysis of Variance) on ranks and Tukey’s post hoc test. Correlations were expressed with the use of Spearman’s correlation. An analysis of the difference in genotype frequency was assessed with the use of χ2 test and Fisher’s exact test. Logistic regression tests were used to determine the independent effect of polymorphism genotypes on the risk of disease. The association was expressed as an OR at 95% CI. In order to verify correlations between the examined parameters, multiple linear regression models were performed. In all instances, *p* < 0.05 was considered statistically significant. Data analyses were performed using the Statistica Software Package, version 13.3 (Polish version; StatSoft, Kraków, Poland).

## 5. Conclusions

1. Oxidative stress in the course of AP contributes to an increase in MT concentration in erythrocyte lysate.
2. MT played an important role in the Cu and Zn homeostasis in both groups, healthy subjects and AP patients, which can be confirmed by a positive correlation between MT and these metals.
3. Healthy subjects and AP patients with the AA genotype for SNP rs11640851 in the *MT1A* gene were the most sensitive to the harmful effects of tobacco smoke xenobiotics, as evidenced by an increase in Cd concentration in the blood.
4. The presence of the CA genotype for SNP rs11640851 in the *MT1A* gene plays an important role in the disturbance of Zn homeostasis in AP patients, especially in smokers.
5. The exposure to tobacco smoke xenobiotics increased the risk of AP occurrence in the subjects with the CC genotype for SNP rs11640851 in the *MT1A* gene by more than fourfold.
6. SNP rs964372 in the *MT1B* gene was associated with an increase in MT concentration in the erythrocytes of AP patients with the GG genotype.
7. The presence of the GC genotype for SNP rs10636 in the *MT2A* gene predisposed the smoking AP patients to a decreased plasma Zn concentration.

## Figures and Tables

**Table 1:** The concentration of metals (Cu, Zn and Cd), MT, Cp and markers of inflammation and oxidative stress in groups of non-smoking and smoking healthy subjects and AP patients. The bold is to distinguish the median values and *p*-values indicating the statistically significance.

	Healthy Subjects			Patients with AP		
**Parameters** **in Erythrocyte Lysate**	**Non-Smokers** **(*n* = 26)**	**Smokers** **(*n* = 25)**	***p***	**Non-Smokers** **(*n* = 17)**	**Smokers** **(*n* = 23)**	*p*
MT [ng/g Hb]	11.7 ± 3.3 (9.5; **10.5**; 13.2)	10.2 ± 2.4 (8.9; **9.6**; 10.8)	0.0764	27.8 ± 7.6 * (23.1; **28.8**; 31.1)	28.5 ± 4.5 ** (25.3; **27.7**; 30.9)	0.7321
SODs [U/g Hb]	157.4 ± 60.0 (114.3; **142.2**; 194.7)	141.4 ± 48.8 (103.7; **140.0**; 176.6)	0.3504	416.1 ± 81.2 * (357.1; **401.1**; 440.8)	429.8 ± 77.9 ** (377.2; **403.9**; 476.4)	0.6613
Cd [µg/L]	0.8 ± 0.3 (0.6; **0.8**; 0.9)	3.1 ± 1.2 (2.3; **3.0**; 3.9)	**<0.0001**	0.6 ± 0.2 (0.5; **0.6**; 0.8)	3.8 ± 1.4 (2.6; **3.2**; 5.5)	**<0.0001**
**Parameters** **in plasma**	**Healthy subjects** **(*n* = 51)**	**AP patients** **(*n* = 40)**	*p*			
MT [ng/mL]	1.8 ± 0.3 (1.6; **1.7**; 1.8)	1.8 ± 0.2 (1.7: **1.7**: 1.8)	0.8049	1.7 ± 0.3 (1.5: **1.6**: 1.9)	1.7 ± 0.2 (1.5: **1.7**: 1.8)	0.5077
Cu [µg/L]	1006.2 ± 128.1 (919.3; **994.6**; 1107.0)	1068.7 ± 160.7 (989.0; **1035.5**; 1210)	0.1520	1063.2 ± 198.6 (955.9; **996.6**; 1014)	1155.6 ± 1180.0 (1080.3; **1161.3**; 1235.6)	0.1476
Zn [µg/L]	936.4 ± 129.0 (840.0; **914.0**; 1010.1)	940.7 ± 142.7 (852.5; **957.2**; 1010.5)	0.9159	759.8 ± 157.0 (684.7; **728.8**; 856.5)	623.8 ± 165.0 ** (487.3; **581.4**; 786.7)	**0.0055**
Cu/Zn ratio	1.1 ± 0.2 (1.0; **1.1**; 1.2)	1.1 ± 0.2 (1.0; **1.1**; 1.2)	0.8018	1.5 ± 0.3 * (1.4; **1.6**; 1.7)	1.7 ± 0.4 ** (1.4; **1.7**; 2.1)	**0.0356**
MDA[nmol/µL]	0.9 ± 0.6 (0.4; **0.7**; 1.5)	0.7 ± 0.3 (0.3; **0.4**; 1.1)	0.2853	2.1 ± 0.7 * (1.7; **2.1**; 2.3)	2.5 ± 0.6 ** (2.2; **2.4**; 2.9)	0.1423
SODs [U/mL]	10.1 ±1.3 (9.1; **10.3**; 11.1)	10.1 ± 1.5 (8.9; **10.1**; 11.3)	0.8814	8.7 ± 2.9(6.5; 8.3; 10.8)	9.1 ± 2.7 (7.3; 8.2; 10.8)	0.4783
TAC [µM CRE]	30.1 ± 15.5 (19.2; **28.6**; 41.1)	27.7 ± 14.9 (17.8: **21.9**; 32.2)	0.7891	942.9 ± 227.2 * (782.6; **898.4**; 1115.6)	867.3 ± 279.1 ** (685.6; **740.5**; 1203.2)	0.4382
Cp [mg/dL]	27.0 ± 13.0 (16.6; **24.2**; 34.6)	32.4 ± 10.5 (24.0; **28.8**; 44.4)	0.1745	20.4 ± 7.2 * (16.9; **18.0**; 23.3)	24.5 ± 7.9 ** (19.6; **25.9**; 28.8)	0.1296
hs-CRP [mg/dL]	0.6 ± 0.3 (0.1; **0.4**; 1.0)	0.5 ± 0.3 (0.2: **0.4**; 0.8)	0.1445	129.1 ± 21.8 * (122.8; **133.6**; 134.7)	141.5 ± 52.3 ** (117.4; 138.5; 190.9)	0.6353
IL-6 [pg/mL]	0.5 ± 0.2 (0.2; **0.4**; 0.6)	0.5 ± 0.2 (0.2; **0.3**; 0.8)	0.8012	46.7 ± 30.8 * (30.9; **31.9**; 74.6)	72.2 ± 29.8 ** (47.0; **73.1**; 97.4)	0.2511

* statistically significant compared to non-smoking healthy subjects. ** statistically significant compared to smoking healthy subjects.

**Table 2:** MT genotype frequency for SNPs rs11640851, rs964372 and rs10636 in patients with acute pancreatitis and healthy subjects.

Polymorphism	Model	Genotype	Healthy Subjects	AP Patients	OR (95% CI)	*p* Value
rs11640851 (*MT1A*)	Codominant	AA CA CC	13 (25.5%) 23 (45.1%) 15 (29.4%)	14 (35%) 18 (45%) 8 (20%)	1.00 1.38 (0.52–3.65) 2.02 (0.64–6.33)	0.48
	Dominant	A/A C/A-C/C	13 (25.5%) 38 (74.5%)	14 (35%) 26 (65%)	1.00 1.57 (0.64–3.89)	0.33
	Recessive	A/A-C/A C/C	36 (70.6%) 15 (29.4%)	32 (80%) 8 (20%)	1.00 1.67 (0.62–4.45)	0.30
	Overdominant	A/A-C/C C/A	28 (54.9%) 23 (45.1%)	22 (55%) 18 (45%)	1.00 1.00 (0.44–2.31)	0.99
rs964372 (*MT1B*)	Codominant	G/G C/G C/C	17 (33.3%) 21 (41.2%) 13 (25.5%)	15 (37.5%) 17 (42.5%) 8 (20%)	1.00 1.09 (0.42–2.80) 1.43 (0.47–4.40)	0.81
	Dominant	G/G C/G-C/C	17 (33.3%) 34 (66.7%)	15 (37.5%) 25 (62.5%)	1.00 1.20 (0.51–2.85)	0.68
	Recessive	G/G-C/G C/C	38 (74.5%) 13 (25.5%)	32 (80%) 8 (20%)	1.00 1.37 (0.50–3.71)	0.54
	Overdominant	G/G-C/C C/G	30 (58.8%) 21 (41.2%)	23 (57.5%) 17 (42.5%)	1.00 0.95 (0.41–2.19)	0.90
rs10636 (*MT2A*)	Codominant	C/C G/C G/G	8 (15.7%) 39 (76.5%) 4 (7.8%)	6 (15%) 29 (72.5%) 5 (12.5%)	1.00 1.01 (0.32–3.23) 0.60 (0.11–3.25)	0.76
	Dominant	C/C G/C-G/G	8 (15.7%) 43 (84.3%)	6 (15%) 34 (85%)	1.00 0.95 (0.30–3.00)	0.93
	Recessive	C/C-G/C G/G	47 (92.2%) 4 (7.8%)	35 (87.5%) 5 (12.5%)	1.00 0.60 (0.15–2.38)	0.46
	Overdominant	C/C-G/G G/C	12 (23.5%) 39 (76.5%)	11 (27.5%) 29 (72.5%)	1.00 1.23 (0.48–3.18)	0.67

**Table 3:** The concentration of metals (Cu, Zn and Cd), MT, Cp and markers of inflammation and oxidative stress in groups of non-smoking and smoking healthy subjects, with regard to genotypes for rs11640851 in the *MT1A* gene. The bold is to distinguish the median values.

Parameters in Erythrocyte Lysate	Non-Smokers			Smokers		
	CA	AA	CC	CA	AA	CC
MT [ng/ g Hb]	9.5; **10.3**; 13.2	9.6; **9.9**; 13.2	9.5; **10.7**; 12.0	8.6; **9.5**; 10.0	9.1; **9.7**; 10.3	9.3; **11.2**; 13.2
SODs [U/g Hb]	94.5; **128.5**; 145.8	115.0; **176.3**; 235.6	132.7; **154.7**; 213.5	112.5; **141.1**; 173.9	81.3; **139.8**; 179.4	67.5; **169.3**; 255.9
Cd [µg/L]	0.6; **0.7**; 0.9	0.7; **0.8**; 0.9	0.7; **0.8**; 0.9	1.4; **2.6**; 3.6 *	2.3; **3.9**; 4.7 **	2.5; **2.6**; 2.6 ***
**Parameters** **in plasma**	**CA**	**AA**	**CC**	**CA**	**AA**	**CC**
MT [ng/L]	1.6; **1.7**; 1.7	1.7; **1.7**; 1.8	1.7; **1.7**; 1.8	1.7; **1.7**; 1.8	1.7; **1.7**; 1.8	1.6; **1.7**; 1.8
Cu [µg/L]	924.9; **996.9**; 1107.0	919.3; **1046.2**; 1092.9	911.2; **990.8**; 1107.9	989.0; **1035.5**; 1241.3	1000.4; **1094.1**; 1232.5	977.7; **994.7**; 1073.3
Zn [µg/L]	822.5; **850.3**; 951.0	803.5; **970.9**; 1012.0	918.0; **1009.7**; 1043.3	891.5; **970.7**; 1001.3	860.4; **944.4**; 1020.0	797.1; **846.1**; 1002.1
Cu/Zn	1.1; **1.2**; 1.4 ***	1.1; **1.1**; 1.2	1.0; **1.0**; 1.1	0.9; **1.1**; 1.2	1.0; **1.1**; 1.2	1.1; **1.1**; 1.2
Cp [mg/dL]	15.9; **31.7**; 39.8	22.4; **26.0**; 40.7	14.7; **19.8**; 23.5	24.4; **31.9**; 46.1	25.4; **26.9**; 30.8	23.6; **34.4**; 43.2
MDA [nmol/µL]	0.5; **0.8**; 1.4	0.3; **0.6**; 1.7	0.4; **0.6**; 1.1	0.2; **0.7**; 1.0	0.2; **0.3**; 0.4	0.7; **1.3**; 1.4
SODs [U/mL]	9.6; **10.3**; 11.3	8.2; **10.2**; 11.1	8.9; **10.2**; 10.7	8.7; **10.1**; 11.6	9.7; **11.2**; 11.4	8.9; **9.3**; 9.5
TAC [µM CRE]	24.7; **28.6**; 48.9	19.2; **19.2**; 19.2	13.3; **28.9**; 41.4	51.7; **51.7**; 51.7	17.8; **25.0**; 32.2	15.1; **18.5**; 21.9
hs-CRP [mg/L]	0.4; **0.5**; 0.8	0.4; **0.6**; 1.7	0.4; **0.5**; 0.7	0.4; **0.5**; 0.7	0.2; **0.3**; 0.4	0.3; **0.5**; 0.7
IL-6 [pg/mL]	0.2; **0.3**; 0.4	0.1; **0.7**; 1.3	0.1; **0.4**; 0.6	0.2; **0.3**; 0.8	0.1; **0.3**; 0.9	0.1; **0.2**; 0.3

* *p* < 0.05 compared to non-smokers with CA genotype. ** *p* < 0.05 compared to non-smokers with AA genotype *** *p* < 0.05 compared to non-smokers with CC genotype.

**Table 4:** The concentration of metals (Cu, Zn and Cd), MT, Cp and markers of inflammation and oxidative stress in groups of non-smoking and smoking patients with AP, with regard to genotypes for rs11640851 in the *MT1A* gene. The bold is to distinguish the median values.

Parameters in Erythrocyte Lysate	Non-Smokers			Smokers		
	CA	AA	CC	CA	AA	CC
MT [ng/ g Hb]	25.7; **30.2**; 31.8	22.6; **24.6**; 29.0	14.4; **23.6**; 32.8	22.9; **25.3**; 30.0	28.4; **29.6**; 34.7	26.8; **29.5**; 33.7
SODs [U/g Hb]	357.1; **413.8**; 440.8	340.2; **383.0**; 431.6	335.1; **434.9**; 534.64	377.3; **403.9**; 456.1	392.5; **400.0**; 556.2	382.8; **431.6**; 476.4
Cd [µg/L]	0.5; **0.8**; 1.0	0.6; **0.7**; 0.8	0.2; **0.2**; 0.3	2.6; **5.7**; 9.8 *	3.3; **4.2**; 5.5 **	2.1; **2.4**; 3.0 ***
**Parameters** **in plasma**	**CA**	**AA**	**CC**	**CA**	**AA**	**CC**
MT [ng/L]	1.5; **1.6**; 1.9	1.6; **1.6**; 1.9	2.0; **2.1**; 2.1	1.5; **1.6**;; 1.8	1.4; **1.7**; 1.9	1.6; **1.7**; 1.9
Cu [µg/L]	912.6; **978.3**; 1007.3	977.9; **1189.0**; 1428.0	987.5; **990.0**; 992.5	1067.2; **1130.9**; 1164.1 *	1231.0; **1240.1**; 1286.3	1093. **1126.3**; 1173.7
Zn [µg/L]	517.2; **600.0**; 718.3	748.1; **834.0**; 870.5	728.8; **883.0**; 1037.1	413.9; **480.5**; 609.6	402.4; **536.1**; 786.7 **	441.2; **526.1**; 603.2
Cu/Zn	1.7; **1.7**; 1.8	1.5; **1.6**; 1.7	1.0; **1.2**; 1.4	1.3; **1.7**; 2.1	1.5; **1.9**; 2.2	1.8; **2.0**; 2.2
Cp [mg/dL]	19.3; **25.0**; 33.5	14.3; **17.2**; 17.9 *	18.0; **19.9**; 21.8	18.6; **22.8**; 26.0	21.3; **27.0**; 31.1 **	24.8; **26.9**; 38.8
MDA [nmol/µL]	1.7; **2.2**; 2.5	2.0; **2.1**; 2.2	1.4; **1.4**; 1.5	2.0; **2.5**; 3.0	2.2; **2.6**; 3.5	2.2; **2.3**; 2.4
SODs [U/mL]	6.3; **8.4**; 13.3	7.2; **8.6**; 10.2	4.7; **6.0**; 7.3	7.3; **8.0**; 10.8	6.8; **9.5**; 11.7	7.3; **8.3**; 10.5
TAC [µM CRE]	771.8; **1107.8**; 1289.6	793.4; **860.7**; 948.6	800.7; **952.0**; 1297.4	700.0; **743.4**; 783.5 *	646.4; **710.0**; 1346.1	496.7; **604.2**; 711.7
hs-CRP [mg/L]	126.8; **130.7**; 134.7	109.9; **128.2**; 145.4	122.5; **127.6**; 132.6	105.4; **151.7**; 193.7	117.4; **154.1**; 190.9	87.6; **135.5**; 138.5
IL-6 [pg/mL]	12.3; **30.9**; 74.6	31.9; **31.9**; 31.9	83.8; **83.8**; 83.8	40.5; **92.7**; 102.1	106.6; **106.6**; 106.6	53.5; **53.5**; 53.5

* *p* < 0.05 compared to non-smokers with the CA genotype, ** *p* < 0.05 compared to non-smokers with the AA genotype, *** *p* < 0.05 compared to non-smokers with the CC genotype.

**Table 5:** The concentration of metals (Cu, Zn and Cd), MT, Cp and markers of inflammation and oxidative stress in groups of non-smoking and smoking healthy subjects, with regard to genotypes for rs964372 in the *MT1B* gene. The bold is to distinguish the median values.

Parameters in Erythrocyte Lysate	Non-Smokers			Smokers		
	CG	GG	CC	CG	GG	CC
MT [ng/ g Hb]	9.5; **10.9**; 16.4	9.4; **10.0**; 11.2	9.5; **10.6**; 11.7	9.2; **9.7**; 11.1	8.6; **9.6**; 9.9	8.5; **9.5**; 12.4
SODs [U/g Hb]	104.2; **120.4**; 141.9	115.1; **138.8**; 145.8	139.0; **166.0**; 194.7	96.0; **141.4**; 169.3	98.7; **126.2**; 164.8	133.1; **160.2**; 179.4
Cd [µg/L]	0.7; **0.8**; 0.9	0.7; **0.8**; 0.9	0.4; **0.6**; 0.9	1.3; **2.6**; 2.6 *	3.6; **3.6**; 4.6 **	2.4; **3.0**; 3.6 ***
**Parameters** **in plasma**	**CG**	**GG**	**CC**	**CG**	**GG**	**CC**
MT [ng/L]	1.6; **1.7**; 1.8	1.6; **1.7**; 1.7)	1.7; **1.8**; 1.8	1.7; **1.7**; 1.8)	1.7; **1.7**; 1.8	1.7; **1.7**; 1.8
Cu [µg/L]	973.3; **1042.1**; 1130.3	830.1; **959.8**; 1018.4	919.3; **984.1**; 1092.9	1001.3; **1057.3**; 1154.9	894.0; **1015.1**; 1245.2	1000.4; **1030.1**; 1094.7
Zn [µg/L]	843.2; **902.5**; 1059.4	909.9; **1001.0**; 1070.1	803.5; **854.5**; 923.8	797.1; **922.2**; 1002.1	839.2; **858.9**; 1019.0	944.4; **983.6**; 1020.0 ***
Cu/Zn	1.1; **1.2**; 1.2	0.9; **1.1**; 1.2	1.0; **1.1**; 1.3	1.0; **1.1**; 1.2	1.0; **1.2**; 1.5	1.0; **1.1**; 1.2
MDA [nmol/µL]	0.4; **0.7**; 1.9	0.6; **0.9**; 1.1	0.3; **0.5**; 1.6	0.2; **0.8**; 1.3	0.3; **0.7**; 1.2	0.3; **0.3**; 0.4
SODs [U/mL]	10.2; **10.8**; 11.4	9.3; **9.8**; 10.5	8.2; **8.9**; 10.5	9.6; **10.2**; 11.2	8.9; **9.3**; 11.2	9.1; **10.3**; 11.4
TAC [µM CRE]	24.7; **48.9**; 55.4	28.6; **34.9**; 41.1	12.0; **16.3**; 24.1	15.1; **23.6**; 32.2	21.9; **36.8**; 51.7	14.5; **17.8**; 17.8
Cp [mg/dL]	17.2; **25.6**; 35.5	13.5; **19.8**; 24.9	19.2; **33.4**; 55.0	23.6; **26.2**; 43.2	24.4; **34.4**; 38.3	25.4; **30.8**; 45.6
hs-CRP [mg/L]	0.4; **0.5**; 0.6	0.4; **0.8**; 0.9	0.4; **0.4**; 0.5	0.4; **0.4**; 0.5	0.3; **0.4**; 0.5	0.2; **0.6**; 0.9
IL-6 [pg/mL]	0.1; **0.4**; 0.6	0.2; **0.6**; 1.5	0.1; **0.2**; 0.7	0.1; **0.2**; 0.3	0.2; **0.3**; 0.5	0.2; **0.5**; 1.0

* *p* < 0.05 compared to non-smokers with CG genotype ** *p* < 0.05 compared to non-smokers with GG genotype *** *p* < 0.05 compared to non-smokers with CC genotype.

**Table 6:** The concentration of metals (Cu, Zn and Cd), MT, Cp and markers of inflammation and oxidative stress in groups of non-smoking and smoking patients with AP, with regard to genotypes for rs964372 in the *MT1B* gene. The bold is to distinguish the median values.

Parameters in Erythrocyte Lysate	Non-Smokers			Smokers		
	CG	GG	CC	CG	GG	CC
MT [ng/ g Hb]	26.1; **29.3**; 31.5	21.3; **22.6**; 25.7	24.6; **27.9**; 31.2	26.6; **27.8**; 29.8	26.9; **32.3**; 35.6 *^,^ **	22.2; **23.3**; 26.8
SODs [U/g Hb]	326.2; **385.9**; 483.1	357.1; **377.5**; 440.8	388.5; **476.6**; 564.8	371.2; **384.9**; 403.9	415.8; **459.6**; 555.0	361.8; **398.5**; 456.1
Cd [µg/L]	0.3; **0.5**; 0.6	0.8; **0.8**; 1.0	0.5; **0.7**; 1.0	2.6; **4.3**; 5.6 ***	2.6; **3.0**; 3.3 *	1.2; **3.6**; 5.9
**Parameters** **in plasma**	**CG**	**GG**	**CC**	**CG**	**GG**	**CC**
MT [ng/L]	1.6; **1.6**; 2.1	1.5; **1.6**; 1.6	1.6; **1.7**; 1.9	1.5; **1.6**; 1.8	1.5; **1.7**; 1.9	1.5; **1.8**; 1.9
Cu [µg/L]	990.0; **996.6**; 1242.9	909.0; **952.3**; 1163.4	1007.1; **1134.4**; 1261.7	1093.4; **1133.7**; 1286.3	1044.1; **1172.6**; 1235.6	1158.6; **1166.1**; 1173.7
Zn [µg/L]	728.8; **811.5**; 856.5	517.2; **617.8**; 718.3	684.8; **784.6**; 884.4	439.3; **506.2**; 572.9 ***	487.3; **564.8**; 786.7	395.0; **413.9**; 480.5
Cu/Zn	1.4; **1.5**; 1.7	1.8; **1.8**; 1.9 ***	1.1; **1.4**; 1.7	1.6; **1.9**; 2.1	1.5; **1.8**; 2.1	1.4; **1.9**; 2.4
MDA [nmol/µL]	1.7; **2.1**; 2.2	1.6; **2.0**; 2.3	1.9; **2.4**; 2.9	2.2; **2.5**; 2.9	2.0; **2.4**; 2.6	2.4; **2.4**; 3.2
SODs [U/mL]	6.3; **6.9**; 7.8	8.7; **11.1**; 13.8	10.2; **10.9**; 11.6	8.2; **9.2**; 11.3	6.8; **8.7**; 10.9	7.1; **7.5**; 7.8
TAC [µM CRE]	739.4; **952.0**; 1289.6	786.3; **830.7**; 1302.8	113.3; **793.4**; 1297.4	673.2; **718.9**; 755.1	659.8; **764.9**; 1308.6	113.3; **711.7**; 1203.2
Cp [mg/dL]	17.5; **18.0**; 19.3	14.3; **19.0**; 26.7	12.3; **15.1**; 17.9	19.4; **24.5**; 30.8 ***	21.3; **26.8**; 29.8	15.3; **25.8**; 26.0
hs-CRP [mg/L]	122.8; **139.9**; 157.1	97.1; **133.6**; 134.7	105.4; **135.0**; 164.6	117.4; **129.5**; 190.9	46.2; **132.6**; 138.5	121.9; **158.3**; 185.9
IL-6 [pg/mL]	30.9; **52.8**; 74.6	24.5; **28.2**; 31.9	53.5; **83.8**; 92.7	40.5; **71.3**; 102.6	12.5; **46.8**; 74.3	53.5; **73.1**; 92.7

* *p* < 0.05 compared to non-smokers with GG genotype ** *p* < 0.05 compared to smokers with CC genotype *** *p* < 0.05 compared to non-smokers with CG genotype.

**Table 7:** The concentration of metals (Cu, Zn and Cd), MT, Cp and markers of inflammation and oxidative stress in the group of non-smoking and smoking healthy subjects, with regard to genotypes for rs10636 in the *MT2A* gene. The bold is to distinguish the median values.

Parameters in Erythrocyte Lysate	Non-Smokers			Smokers		
	GC	CC	GG	GC	CC	GG
MT [ng/ g Hb]	9.5; **10.5**; 15.1	9.5; **10.1**; 10.7	8.0; **13.3**; 18.6	9.0; **9.5**; 10.0 *	8.6; **8.8**; 11.0	12.4; **14.8**; 17.2
SODs [U/g Hb]	138.8; **145.8**; 218.7	92.0; **94.5**; 115.1	104.2; **178.4**; 252.5	103.7; **140.1**; 184.5	81.3; **112.5**; 142.4	104.2; **139.0**; 173.9
Cd [µg/L]	0.5; **0.7**; 0.8	0.7; **0.9**; 1.6	0.8; **0.9**; 0.9	2.3; **3.0**; 3.9 *	1.3; **3.6**; 4.7	2.6; **3.3**; 4.0
**Parameters** **in plasma**	**GC**	**CC**	**GG**	**GC**	**CC**	**GG**
MT [ng/L]	1.6; **1.7**; 1.8	1.7; **1.7**; 1.7)	1.7; **1.8**; 1.9	1.7; **1.7**; 1.8	1.6; **1.7**; 1.8	1.7; **1.7**; 1.8
Cu [µg/L]	920.7; **1018.4**; 1100.0	868.5; **924.9**; 1018.4	986.9; **1048.8**; 1110.7	989.0; **1034.3**; 1210.6	687.6; **1024.9**; 1232.5	1011.8; **1052.9**; 1094.1
Zn [µg/L]	838.5; **918.0**; 1075.4	854.2; **902.0**; 992.3	895.4; **933.1**; 970.9	839.2; **920.4**; 1002.1	924.0; **1019.0**; 1337.0	970.1; **985.9**; 1001.7
Cu/Zn	1.0; **1.2**; 1.3	1.0; **1.0**; 1.2	1.1; **1.1**; 1.2	1.0; **1.1**; 1.2	0.9; **1.0**; 1.1	1.0; **1.1**; 1.1
MDA [nmol/µL]	0.3; **0.6**; 0.9	1.2; **1.9**; 2.2 *	1.6; **1.9**; 2.2	0.3; **0.4**; 1.3	0.2; **0.7**; 1.2	0.2; **0.3**; 0.3
SODs [U/mL]	8.9; **10.2**; 11.1	9.7; **10.3**; 10.5	8.7; **10.0**; 11.4	9.1; **9.9**; 11.2	8.3; **10.3**; 11.9	8.7; **10.0**; 11.4
TAC [µM CRE]	19.2; **28.6**; 41.1	27.9; **28.7**; 29.8	36.2; **36.2**; 36.2	17.8; **21.9**; 32.2	21.9; **26.5**; 39.6	14.5; **27.8**; 37.1
Cp [mg/dL]	19.8; **26.4**; 39.8	14.0; **15.9**; 19.2	14.1; **19.9**; 25.6	19.8; **26.4**; 39.8	14.0; **15.9**; 19.2	14.1; **19.9**; 25.6
hs-CRP [mg/L]	0.4; **0.5**; 0.7	0.5; **0.8**; 1.3	0.4; **0.5**; 0.5	0.3; **0.4**; 0.7	0.4; **0.5**; 0.5	0.2; **0.4**; 0.5
IL-6 [pg/mL]	0.2; **0.4**; 0.6	0.1; **0.4**; 0.7	0.1; **0.7**; 1.3	0.2; **0.3**; 0.5	0.8; **0.9**; 0.9	0.3; **0.5**; 0.6

* *p* < 0.05 compared to non-smokers with GC genotype.

**Table 8:** The concentration of metals (Cu, Zn and Cd), MT, Cp and markers of inflammation and oxidative stress in the group of non-smoking and smoking patients with AP, with regard to genotypes for rs10636 in the *MT2A* gene. The bold is to distinguish the median values.

Parameters in Erythrocyte Lysate	Non-Smokers			Smokers		
	GC	CC	GG	GC	CC	GG
MT [ng/ g Hb]	22.5; **27.1**; 30.6	22.6; **27.2**; 31.8	29.0 **30.9**; 32.8	24.2; **26.8**; 29.8	25.7; **28.2**; 33.6	33.7; **34.7**; 36.6 *
SODs [U/g Hb]	357.1; **401.1**; 431.6 *	377.5; **409.2**; 440.8	312.3; **423.5**; 534.6	371.2; **398.5**; 440.2	391.4; **439.5**; 516.4	476.4; **532.4**; 588.4
Cd [µg/L]	0.5; **0.6**; 0.8	0.8; **0.9**; 1.0	03; **0.5**; 0.8	2.5; **3.6**; 5.6 **	3.0; **3.4**; 3.9	2.1; **2.7**; 3.3
**Parameters** **in plasma**	**GC**	**CC**	**GG**	**GC**	**CC**	**GG**
MT [ng/L]	1.6; **1.7**; 1.9	1.4; **1.5**; 1.6	1.6; **1.7**; 1.9	1.5; **1.6**; 1.8	1.61; **1.7**; 1.8	1.4; **1.6**; 2.0
Cu [µg/L]	948.7; **996.6**; 1007.1	955.9; **1163.4**; 1370.9	987.5; **1236.3**; 1485.1	1158.6; **1173.7**; 1240.1 **	952.6; **1073.6**; 1235.7	1067.2; **1093.4**; 1231.0
Zn [µg/L]	684.7; **723.6**; 811.5	517.2; **625.5**; 733.8	856.5; **946.8**; 1037.1	395.0; **445.1**; 536.1 **, ***	564.4; **733.6**; 866.6	553.2; **670.0**; 786.7
Cu/Zn	1.4; **1.5**; 1.7	1.8; **1.9**; 1.9	1.0; **1.3**; 1.7	1.6; **2.1**; 2.2 **	1.3; **1.6**; 1.9	1.4; **1.8**; 2.2
MDA [nmol/µL]	1.9; **2.1**; 2.3	1.6; **2.0**; 2.5	2.0; **2.1**; 2.2	2.0; **2.3**; 2.8	2.4; **2.5**; 3.0	2.6; **3.2**; 3.7
SODs [U/mL]	6.9; **8.4**; 10.0	11.4; **12.8**; 14.2	4.7; **5.5**; 6.2	7.3; **8.2**; 10.9	6.5; **9.1**; 11.2	6.8; **8.9**; 9.4
TAC [µM CRE]	793.4; **1107.7**; 1297.4	771.8; **786.3**; 806.7	798.6; **950.3**; 1019.7	673.2; **734.1**; 1228.9	446.4; **867.4**; 1167.3	496.7; **659.8**; 685.6
Cp [mg/dL]	14.3; **18.0**; 23.3	19.0; **22.9**; 26.7	17.5; **18.5**; 19.5	19.4; **25.9**; 28.2	19.6; **26.8**; 29.8	12.0; **21.3**; 33.8
hs-CRP [mg/L]	122.8; **133.6**; 134.7	97.1; **114.3**; 131.6	106.5; **131.8**; 157.1	123.4; **138.5**; 184.5	42.6; **87.6**; 132.6	124.6; **127.7**; 130.9
IL-6 [pg/mL]	30.9; **31.9**; 74.6	24.5; **24.5**; 24.5	29.3; **29.3**; 29.3	47.0; **73.1**; 97.4	40.5; **71.3**; 102.6	74.3; **74.3**; 74.3

* *p* < 0.05 compared to smokers with GC genotype ** *p* < 0.05 compared to non-smokers with GC genotype *** *p* < 0.05 compared to smokers with CC genotype.

**Table 9:** Clinical characteristics of the participants in the study.

Parameters	AP Patients	
	Non-Smokers Mean ± SD	Smokers Mean ± SD
Ranson Criteria [score]	2.66 ± 1.03	2.52 ± 0.69
Lipase [U/l]	576.71 ± 393.13	692.00 ± 268.95
Erythrocytes [10^12^/L]	3.86 ± 0.66	4.16 ± 0.94
Leukocytes [10^9^/L]	10.24 ± 4.49	10.55 ± 4.41
Hemoglobin [g/dL]	11.24 ± 1.86	11.79 ± 2.15
Hematocrit [%]	35.49 ± 4.42	36.36 ± 6.22
Bilirubin (total) [mg/dL]	1.26 ± 0.57	0.99 ± 0.61
Alkaline phosphatase [U/L]	69.17 ± 19.02	77.75 ± 22.34
Glucose [mg/dL]	90.17 ± 19.49	94.86 ± 17.47
Urea [mg/dL]	32.33 ± 18.55	19.43 ± 8.71
Creatinine [mg/dL]	0.75 ± 0.09	0.97 ± 0.83
Cotinine [ng/mL]	3.3 ± 7.9	104.3 ± 61.4 ^1)^
Pack-year [pack of cigarettes per day × smoking time]	not applicable	25.7 ± 21.5

^1)^ *p* < 0.05 compared to non-smokers.

**Table 10:** The conditions of PCR reactions for polymorphisms in MT genes (*MT1A, MT1B, MT2A*).

SNP	Gene	Chromosome/ Chromosome Position	Genotype	Starters Sequency 5′-3′ (Contents of Nucleotides Bases)	Melting Temperature [°C]	Annealing Temperature [°C]	The Content of GC Pairs [%]
rs11640851	*MT1A*	16/56639315	A/C	F: AAGGGGGAAGTGGACACTCA (20)	60.4	58	55.0
				R: GTCAGGAGACAACTGGTGGA (20)	59.0		55.0
rs964372	*MT1B*	16/56652118	C/G	F: CACAGTGTCCCTGGGTTAG (19)	57.1	57	57.9
				R: TAGGTGGGTGACATGGAGC (19)	58.7		57.9
rs10636	*MT2A*	16/56609431	C/G	F: CCGCTCCCAGATGTAAAGAA (20)	57.3	55	50.0
				R: GGCATATAAAGAAAACCAGAGACA(24)	57.0		37.5

**Table 11:** The digestion conditions of PCR products with restriction enzyme.

SNP	Gene	Product Length	Restriction Enzymes, Company, Catalog Number, Recognized Sequence	Cuts Temperature [°C]	Genotype, Fragments After Restrictions
rs11640851	*MT1A*	367 bp	MnII Thermo Fisher Scientific Cat. No. ER1072	37	CA: 191 bp, 145 bp, 110 bp, 66 bp, 46 bp AA: 191bp, 110 bp, 66 bp CC: 145 bp. 110 bp, 66 bp, 46 bp
			5′ CCTCN_7_↓ 3′ 3′ GGAGN_6_↑5′		
rs964372	*MT1B*	180 bp	HaeIII Thermo Fisher Scientific Cat. No. ER0151	37	CG: 86 bp, 94 bp, 180 bp AA:86 bp, 94bp CC: 180 bp
			5′ GG↓CC 3′ 3′ CC↑GG 5′		
rs10636	*MT2A*	157 bp	MaeIII	55	GC: 63 bp, 95 bp, 157 bp CC: 63 bp, 95 bp GG: 157 bp
			Sigma-Aldrich, Cat. No. 10822230001 5′ ↓GTNAC 3′ 3′ CANTG↑ 5′		

## Data Availability

Data available on request due to privacy.

## Supplementary Materials

The following are available online at https://www.mdpi.com/article/10.3390/ijms22115725/s1.
