# Supplementary material for: Importance of Genetic Polymorphisms in MT1 and MT2 Genes in Metals Homeostasis and Their Relationship with the Risk of Acute Pancreatitis Occurrence in Smokers—Preliminary Findings
Source: Int J Mol Sci. 2021 May 27;22(11):5725. doi: 10.3390/ijms22115725 (PMC8197913; doi:10.3390/ijms22115725)
Supplement: Supplementary file 1 [file ijms-22-05725-s001.zip › ijms-1222439-supplementary.pdf]

Table S1. The concentration of metals (Cu, Zn), MT, Cp and markers of inflammation and oxidative stress in the group AP patients in terms of etiology of disease

Parameters in erythrocyte lysate	Biliary	Alcohol	Alcohol and hyperlipidaemia	Hyperlipidaemia	Other
**MT [ng/g Hb]**	28.3 ±4.7 (23.7; 30.2; 31.8)	27.5 ±4.7 (23.7; 26.9; 29.6)	28.7 ±4.1 (26.4; 28.6; 30.8)	27.3 ±7.6 (21.1; 25.7; 33.6)	26.0 ±6.0 (23.3; 26.9; 29.7)
**SODs [U/g Hb]**	421.4 ±99.5 (340.2; 389.6; 534.6)	459.3 ±82.3 (392.5; 440.2; 556.2)	392.1 ±72.2 (332.6; 371.2; 472.5)	436.5 ±103.6 (357.1; 398.5; 553.7)	398.1 ±66.3 (8.0; 9.4; 10.7)
**Parameters in plasma**	**Biliary**	**Alcohol**	**Alcohol and hyperlipidaemia**	**Hyperlipidaemia**	**Other**
**MT [ng/ml]**	1.9 ±0.2 (1.8; 1.9; 2.2)	1.6 ±0.2 (1.4; 1.5; 1.8)	1.7 ±0.2 (1.5; 1.7; 1.9)	1.6 ±0.2 (1.5; 1.5; 1.5)	1.8 ±0.2 (1.6; 1.7; 1.8)
**Cu [µg/l]**	1027.3 ±90.0 (971.7; 997.3; 1082.8)	1206.3 ±135.4 (1067.2; 1240.1; 1286.3)	1214.8 ±104.3 (1103.3; 1231.0; 1310.1)	876.8 ±10.6 (869.3; 876.8; 884.3)	1078.6 ±106.6 (992.5; 1073.6; 1164.1)
**Zn [µg/l]**	720.8 ±238.4 (517.2; 684.7; 884.4)	542.6 ±182.3 (363.6; 506.2; 733.8)	494.8 ±102.7 (408.2; 483.6; 581.4)	772.0 ±75.9 (718.3; 772.0; 825.7)	646.8 ±128.5 (564.8; 641.5; 728.8)
**Cu/Zn ratio**	1.6 ±0.5 (1.1; 1.6; 1.8)	1.9 ±0.5 (1.4; 1.9; 2.3)	1.9 ±0.5 (1.7; 2.1; 2.2)	1.6 ±0.4 (1.3; 1.7; 1.8)	1.8 ±0.3 (1.5; 1.7; 2.1)
**MDA[nmol/µL]**	2.4 ±1.1 (1.9; 2.0; 2.4)	2.5 ±0.7 (2.0; 2.3; 3.1)	2.5 ±0.6 (2.2; 2.6; 2.9)	2.1 ±0.4 (2.0; 2.3; 2.4)	2.2 ±0.5 (1.8; 2.2; 2.5)
**SODs [U/ml]**	9.1 ±2.7 (7.8; 8.8; 10.2)	10.2 ±3.8 (7.2; 9.7; 11.5)	9.0 ±1.1 (8.2; 8.9; 9.5)	9.4 ±3.0 (6.9; 8.4; 12.0)	9.3 ±1.5 (8.0; 9.4; 10.7)
**TAC [µM CRE]**	1125.5 ±329.2 (870.2; 1077.6; 1288.4)	961.5 ±329.1 (659.8; 800.7; 1308.6)	773.0 ±475.6 (685.6; 734.1; 769.8)	808.7 ±684.5 (371.7; 692.9; 1245.6)	863.3 ±241.5 (709.0; 783.5; 988.4)
**Cp [mg/dl]**	19.5 ±5.6 (15.3; 18.7; 25.1)	25.1 ±8.2 (19.0; 26.3; 31.2)	25.0 ±7.5 (20.2; 23.5; 26.0)	21.9 ±11.6 (12.2; 27.1; 29.8)	19.5 ±4.2 (16.9; 18.3; 22.2)
**hs-CRP [mg/dl]**	116.6 ±12.8 (105.9; 114.6; 127.2)	151.6 ±48.4 (110.8; 157.7; 192.3)	139.0 ±26.8 (123.4; 130.2; 154.5)	134.7 ±0.0 (134.7; 134.7; 134.7)	97.3 ±51.5 (42.6; 132.6; 133.6)
**IL-6 [pg/ml]**	83.7 ±9.0 (74.6; 83.8; 92.7)	53.5 ±53.5 (53.5; 53.5; 53.5)	71.3 ±43.5 (40.5; 71.3; 102.1)	55.4 ±35.6 (41.2; 56.3; 87.6)	38.9 ±9.9 (31.9; 38.9; 45.9)

The data were presented as mean ±standard deviation and upper quartile, median, lower quartile.

Table S2. The concentration of metals (Cu, Zn), MT, Cp and markers of inflammation and oxidative stress in the group of healthy subjects and AP patients in terms of sex.

	**Healthy subjects**			**Patients with AP**		
**Parameters in erythrocyte lysate**	**Women (n=26)**	**Men (n=25)**	**p**	**Women (n=13)**	**Men (n=27)**	**p**
MT [ng/g Hb]	11.5 ±3.2 (9.5: **10.3**; 11.9)	10.2 ±2.3 (8.6; **9.5;** 10.7)	0.1445	28.2 ±5.2 * (25.6; **27.9**; 30.7)	28.9 ±6.6 ** (24.9; **28.0**; 31.4)	0.1837
SODs [U/g Hb]	158.7 ±54.3 (114.3; **144.2**; 190.9	140.5 ±57.0 (96.0; **133.1**; 165.3)	0.2965	422.2 ±92.3 * (332.6; **431.6**; 479.0	417.9 ±74.0 ** (374.2; **399.3**; 448.1)	0.8877
**Parameters in plasma**	**Women (n=26)**	**Men (n=25)**	**p**	**Women (n=26)**	**Men (n=25)**	**p**
MT [ng/ml]	1.8 ±0.3 (1.7; **1.7;** 1.8)	1.7 ±0.1 (1.6; **1.7**; 1.7)	0.1134	1.6 ±0.2 * (1.5; **1.6**; 1.9)	1.7 ±0.2 (1.5; **1.7**; 1.8)	0.3395
Cu [µg/l]	1056.8 ±126.1 (977.7; **1038.8**; 1110.7)	1001.2 ±179.0 (838.4; **991.9**; 1089.5)	0.2260	1053.6 ±187.7 (952.3; **1003.9;** 1073.6)	1149.6 ±137.0 ** (1067.2; **1161.3;** 1240.1)	0.8132
Zn [µg/l]	911.2 ±142.6 (838.9; **898.7**; 992.7)	1000.2 ±84.7 (970.9; **1014.3**; 1043.3)	**0.0358**	672.3 ±167.7 * (517.2; **663.1;** 825.7)	664.5 ±145.6 ** (544.7; **664.0**; 757.8)	0.1541
Cu/Zn ratio	1.2 ±0.2 (1.0; **1.2**; 1.3)	1.0 ±0.1 (0.9; **1.0**; 1.1)	**0.0012**	1.6 ±0.3 * (1.3; **1.7;** 1.8)	1.7 ±0.4 ** (1.3; **1.6;** 2.1)	0.6183
MDA [nmol/µl]	0.9 ±0.6 (0.4; **0.7**; 1.3)	0.7 ±0.6 (0.2; **0.4**; 0.9)	0.1755	2.8 ±1.0 * (2.2; **2.5;** 3.2)	2.9 ±1.1 ** (2.2; **2.7**; 3.4)	0.1538
SODs [U/ml]	10.3 ±1.5 (9.0; **10.4;** 11.4)	9.7 ±0.9 (9.0; **9.7**; 10.4)	0.2215	9.4 ±2.3 (7.7; **9.5;** 10.8)	8.6 ±2.9 ** (7.1; **8.0;** 9.8)	0.3834
TAC [µM CRE]	29.0 ±16.0 (16.4; **23.3**; 45.0)	34.7 ±7.4 (28.9; **32.2;** 43.0)	0.5679	869.9 ±391.8 * (708.9; **782.6;** 988.4)	895.8 ±361.6 ** (692.8; **854.8**; 1211.4)	0.7678
Cp [mg/dl]	31.3 ±12.8 (22.2; **30.8;** 403)	23.9 ±8.5 (17.8; **24.7;** 26.0)	0.0726	23.5 ±7.2 (17.5; **25.8**; 26.8)	22.5 ±7.8 (17.9; **20.8**; 28.2)	0.7233
hs-CRP [mg/dl]	0.5 ±0.2 (0.4; **0.5**; 0.7)	0.6 ±0.3 (0.4; **0.5;** 0.7)	0,1975	116.5 ±50.2 * (80.0; **132.1;** 145.0)	134.4 ±30.2 ** (114.6; **130.2**; 138.5)	0,5828
IL-6 [pg/ml]	0.5 ±0.4 (0.2; **0.3;** 0.6)	0.6 ±0.3 (0.4; **0.5;** 0.7)	0,8178	63.4 ±27.7 * (31.9; **74.6**; 83.8)	54.0 ±32.5 ** (30.9; **45.9;** 92.7)	0,1779

The data were presented as mean ±standard deviation and upper quartile, median, lower quartile. *statistically significant compared to healthy women ** statistically significant compared to healthy men

Table S3. Correlation coefficients for the group of healthy subjects and AP patients in terms of rs11640851 in MT1A gene.

Correlated parameters	r	p
**NON-SMOKING HEALTHY SUBJECTS**		
**Individuals with the CA genotype:**		
Cu [µg/l] : Cp [mg/l]	0.1909	0.0005
Cu/Zn : Cp [mg/l]	0.6273	0.0359
**Individuals with the AA genotype:**		
Cu/Zn : MT [ng/l]	0.9000	0.0374
**SMOKING HEALTHY SUBJECTS**		
**Individuals with the CC genotype:**		
MT [ng/g Hb] : Cp [mg/l]	0.9000	0.0473
**NON-SMOKING PATIENTS WITH ACUTE PANCREATITIS**		
**Individuals with the CA genotype:**		
MT [ng/g Hb] : Zn [µg/l]	0.9000	0.0384
**Individuals with the AA genotype:**		
Cp [mg/l] : Cu/Zn	0.9000	0.0370
**SMOKING PATIENTS WITH ACUTE PANCREATITIS**		
**Individuals with the CA genotype:**		
MT [ng/l] : Cu [µg/l]	0.8286	0.0416
**Individuals with the AA genotype:**		
MT [ng/g Hb]: Zn [µg/l]	0.9000	0.0474
**Individuals with the CC genotype:**		
Cp [mg/l] : TAC [µM CRE]	-0.8858	0.0188

Table S4. Correlation coefficients for the group of healthy subjects and AP patients in terms of rs964372 in MT1B gene.

Correlated parameters	r	p
**NON-SMOKING HEALTHY SUBJECTS**		
**Individuals with the GG genotype:**		
Cu [µg/l] : Cp [mg/l]	0.8857	0.0188
Cu/Zn : Cp [mg/l]	0.8214	0.0235
**Individuals with the CC genotype:**		
Cu [µg/l] : Cp [mg/l]	0.8286	0.0415
**SMOKING HEALTHY SUBJECTS**		
**Individuals with the CG genotype:**		
MT [ng/l] : Cu [µg/l]	0.7381	0.0366
**Individuals with the CC genotype:**		
Cp [mg/l] : MT [ng/l]	0.8286	0.0415
Cp [mg/l] : Cu/Zn	0.9529	0.0048
**SMOKING PATIENTS WITH ACUTE PANCREATITIS**		
**INDIVIDUALS WITH THE CG GENOTYPE:**		
Cu/Zn : MDA [µmol/l]	0.7676	0.0036

Table S5. Correlation coefficients for the group of healthy subjects and AP patients in terms of rs10636 in MT2A gene.

Correlated parameters	r	p
**NON-SMOKING HEALTHY SUBJECTS**		
**Individuals with the GC genotype:**		
MT [ng/g Hb] : Cd [µg/l]	0.6225	0.0132
Cp [mg/l] : Cu [µg/l]	0.5750	0.0249
Cp [mg/l] : IL-6 [ng/l]	0.6606	0.0269
**Individuals with the CC genotype:**		
MT [ng/l] : Cu [µg/l]	0.9000	0.0374
**SMOKING HEALTHY SUBJECTS**		
**Individuals with the GC genotype:**		
Cp [mg/l] : MT [ng/l] :	0.5889	0.0306
Cp [mg/l] : Cu [µg/l]	0.6593	0.0140
